# Evaluation of a smartphone application for self-help for patients with social anxiety disorder: a randomized controlled study—SMASH

**DOI:** 10.1186/s13063-023-07168-5

**Published:** 2023-03-01

**Authors:** Jan Marius Schittenhelm, Christoph von Borell, Celina Clément, Johanna Schüller, Ulrich Stangier, Juergen Hoyer

**Affiliations:** 1 Institute of Psychology, Goethe-Universitaet Frankfurt, Varrentrappstr. 40-42, 60486 Frankfurt, Germany; 2 Institute for Clinical Psychology and Psychotherapy, Technische Universitaet Dresden, Dresden, Germany

**Keywords:** Social anxiety disorder, ICBT, App-based treatment, Cognitive behavioral therapy, Social phobia, App

## Abstract

**Background:**

There is growing evidence that Internet-based cognitive behavioral therapy (ICBT) is as effective as a stand-alone treatment and helps facilitating access to treatment. Given the complexity of the treatment, we argue that the effect of ICBT could be even greater if guided by a therapist, as this could increase treatment adherence. We modified an established and well-evaluated treatment approach and developed a mobile application for treating social anxiety disorder (SAD). In the present study, we compare the efficacy of app use alone (APP) with video-based, therapist-guided app use (TG-APP) and with a wait-list control group (WLC) in terms of symptom reduction, and various secondary outcomes such as increase in quality of life or decrease of general psychological distress.

**Methods/design:**

A within-between interaction design with randomization to one of three conditions will be used. In the APP condition, patients receive only the app without any additional contact with therapists, while in the TG-APP condition, therapists provide 8 sessions of video-based treatment in addition to using the app. The study will be conducted in two university outpatient treatment centers with reliably diagnosed SAD patients. The primary outcome will be defined as change in SAD symptoms, as measured by the Liebowitz Social Anxiety Scale (expert rating). Furthermore, a wide range of self-reports and clinician ratings for other symptoms (depression, general psychopathology) or quality of life will be used. A simulation-based power analysis for a 3 × 2 interaction effect (group × time) on the primary outcome in a linear mixed model resulted in a total sample size of *N* = 165.

**Discussion:**

The present study will be one of the first to examine the additional benefit of therapist-guided video sessions regarding the use of an app treating SAD. Study results are pivotal to future treatment application in SAD.

**Supplementary Information:**

The online version contains supplementary material available at 10.1186/s13063-023-07168-5.

## Administrative information

**Table:** 

Title	Evaluation of a smartphone application for self-help for patients with social anxiety disorder: a randomized controlled study (SMASH)
Trial registration.	Trial registration: ClinicalTrials.gov, NCT05554718. Registered 16 September 2022, https://clinicaltrials.gov/show/NCT05554718. The pilot study was registered in the German Clinical Trials Register (DRKS), DRKS00029701. Registered 01 August 2022. https://www.drks.de/drks_web/navigate.do?navigationId=trial.HTML&TRIAL_ID=DRKS00029701
Protocol version	Version 2 of 16-12-2022
Funding	This study is funded by Mindable Health GmbH, Germany. Contact: team@mindable.health
Author details	J. M. Schittenhelm: Goethe Universitaet Frankfurt, Germany C. von Borell: Technische Universitaet Dresden, Germany C. Clément: Goethe Universitaet Frankfurt, Germany J. Schüller: Goethe Universitaet Frankfurt, Germany U. Stangier: Goethe Universitaet Frankfurt, Germany J. Hoyer: Technische Universitaet Dresden, Germany
Name and contact information for the trial sponsor	Prof. Dr. Jürgen Hoyer (Principal Investigator): juergen.hoyer@tu-dresden.de; Prof. Dr. Ulrich Stangier (Principal Investigator): stangier@psych.uni-frankfurt.de
Role of sponsor	The funders do not have a role in collection, analysis, and interpretation of data.

## Introduction

Social anxiety disorder (SAD) is characterized by the fear of behaving in a way that is negatively perceived by others [1]. In the USA, the disorder has a lifetime prevalence of 10.7% and a 12-month prevalence of 7.4% [2]; in Europe, the estimated lifetime prevalence is 6.7% [3]. The disorder is associated with high psychosocial impairment and disability [3].

On the one hand, there are ways to treat SAD. For example, a recent meta-analysis demonstrated that individualized cognitive therapy (CT) based on the model of Clark and Wells [4] was among the most effective treatments, outperforming pharmacotherapy and other psychotherapeutic approaches in several studies [5]. On the other hand, although treatment is recommended in international guidelines, access to effective treatment for SAD patients remains difficult, in part because SAD-related anxiety and impairment exacerbate difficulties and delays in initial treatment contact [6–8].

Addressing these treatment barriers, Lee and Stapinski [9] found evidence that social anxiety is related to a perception of decreased risk of negative evaluation and greater control in online communication. Therefore, Internet-delivered cognitive-behavioral treatments (ICBTs) may be a promising approach to the treatment of SAD. ICBTs may reduce the threshold to seek treatment and provide cost-efficient [10, 11] and highly scalable access to scientifically evaluated treatment programs [12]. Recent empirical data shows that internet-based cognitive behavioral treatments can be as effective as traditional face-to-face therapy [12, 13] and also reach favorable long-term outcomes [12, 14]. Stott et al. [15] for example were able to show that an online treatment program, based on the Clark and Wells approach, offered through a website, was significantly reducing SAD-related symptoms.

Previous studies that have examined the effectiveness of ICBTs have largely used treatment programs delivered via a computer. Today, however, the use of a smartphone including its apps is even more widespread, rendering mobiles a promising option for the treatment of mental disorders. It might even have more advantages than ICBTs delivered via a computer, as apps seem to be easily integrated into daily routines [16] and are convenient for both the patient and the practitioner [17]. Furthermore, using a mobile phone has the advantage that patients can use protocols or other forms of assessments when the situation occurs so that some forms of perception and reporting biases can be minimized [18]. As an example, Stolz et al. [16] have been able to show that an app based on the Clark and Wells [4] treatment approach with text-based guidance during the process can significantly reduce symptoms of social anxiety. However, it remains unclear whether their app would also work without additional guidance.

This raises the important research question of whether the effectiveness of apps can be enhanced by accompanying face-to-face sessions with a therapist. To date, few studies have addressed this question. The authors assume that therapist guidance could additionally increase compliance with ICBT treatment as well as the motivation to complete it. Another advantage of therapist-guided ICBT over nonguided ICBT could be higher patient safety, as the therapist can assist when questions or ambiguities arise, or when adverse side effects of ICBT occur, such as worsening of symptoms, negative well-being, or noncompliance [19].

Particularly as a result of the COVID-19 pandemic, new ways of providing therapeutic support have been developed: Due to the limitations of physical contact, the use of videoconferencing has increased dramatically. A meta-analysis has shown that video-based psychotherapy typically has large effect sizes and that the difference to in-person therapy is negligible [20]. The use of this modality to treat mental disorders could be an important step in increasing accessibility to psychotherapy, not only because of the ongoing (or possibly recurring) pandemic situation, but also to overcome other barriers to in-person treatment, such as illnesses that limit the scope of activities, specialized treatment programs that exist only in distant locations, or difficulty finding a therapist in rural areas.

In summary, the use of ICBT delivered via a smartphone application could improve the accessibility of psychotherapy and integrate interventions into everyday life, making it a promising approach for the future treatment of SAD. However, few studies have attempted to implement Clark and Wells’ [4] well-evaluated approach into a treatment program in form of an app, and even fewer have examined whether the app works without further guidance. Therefore, our goal was to develop and evaluate an app that could effectively help patients without therapeutic support. In addition, questions remain about how the app and face-to-face therapy can be combined and how this combined treatment compares to using the app alone. We aim to investigate these questions in a randomized controlled trial focusing on (1) the overall efficacy of a newly developed app for SAD and (2) the potential benefit of additional therapeutic support delivered via videoconferencing.

### Objectives

The main goal of the present study is to evaluate whether the use of the app-based intervention helps to decrease symptoms of social anxiety disorder and has positive effects on mental health. There will be two experimental treatment conditions: App-based CT (APP) and app-based CT plus 8 therapist-guided video-delivered therapy sessions (TG-APP). Both will be compared to a wait-list control group (WLC). The therapists will be trained in Cognitive Therapy based on the model of Clark and Wells [4], which has been proven to be effective in face-to-face therapy [5] and in ICBT [15]. In a randomized controlled trial (RCT), the following hypotheses will be tested:

- Hypothesis 1a: In the APP and TG-APP condition, SAD-related symptoms, as measured by the LSAS, will decrease significantly more from baseline to post-treatment than in the WLC condition.
- Hypothesis 1b: The TG-APP condition results in a significantly greater reduction in SAD-related symptoms, as measured by the LSAS, than the APP and the WLC conditions, from baseline to post-treatment.
- Hypothesis 1c: In the APP and the TG-APP condition, there will be no significant deterioration of SAD-related symptoms, as measured by the LSAS, from post-treatment to follow-up.
- Hypothesis 2a: In the APP and TG-APP conditions, significant improvements in secondary outcome variables, including social phobic cognitions, depression symptoms, general psychological distress, psychological impairment, quality of life, and interpersonal pleasure, are achieved from baseline to post-treatment compared to the WLC condition.
- Hypothesis 2b: In the TG-APP condition, improvements in secondary outcome variables from baseline to post-treatment will be significantly larger than in the APP condition.
- Hypothesis 2c: In the APP and TG-APP condition, there will be no significant deterioration in secondary outcome variables from post-treatment to follow-up.
- Hypothesis 3: Individual learning styles contribute significantly to predicting success in both treatment conditions.
- Hypothesis 4: The increase in social approach behavior will mediate the effect of the TG-APP versus APP condition.

### Trial design

The design of the planned bi-centric randomized controlled superiority trial will be based on a within-between interaction group design with three groups (WLC, APP, TG-APP) and parallel group assignment. The primary outcome criterion is the change in symptom severity of SAD, as measured by the Liebowitz Social Anxiety Scale (LSAS) [21, 22]. It will be assessed by trained, blinded, independent raters at pre-treatment, post-treatment, and 6 months after post-treatment (follow-up). The planned allocation ratio for TG-APP vs. APP only vs. WLC will be 1:1:0.7.

## Methods

### Design and sample size

We conducted a simulation-based a priori power analysis for a 3 × 2 interaction (group ( time) in a linear mixed model for the primary outcome of SAD symptom severity (according to the LSAS), using the R package “simr” [23]. The analysis is based on a model including a random intercept for participants, a moderate effect size of Cohen’s *d* = 0.5 for the treatment effect in the APP condition, and a large treatment effect of Cohen’s *d* = 0.8 for the TG-APP condition compared to the control group (implying a small effect in favor of TG-APP between both treatment conditions).

We chose these effect sizes as conservative estimates based on the results of a recent meta-analysis reporting moderate effect sizes in ICBTs for SAD [12]. Comparable to Stolz et al. [16], we decided to recruit different sample sizes for the control and treatment conditions, because treatment effects compared to the control group were assumed to be larger than between the two treatment conditions.

**Fig. 1:**
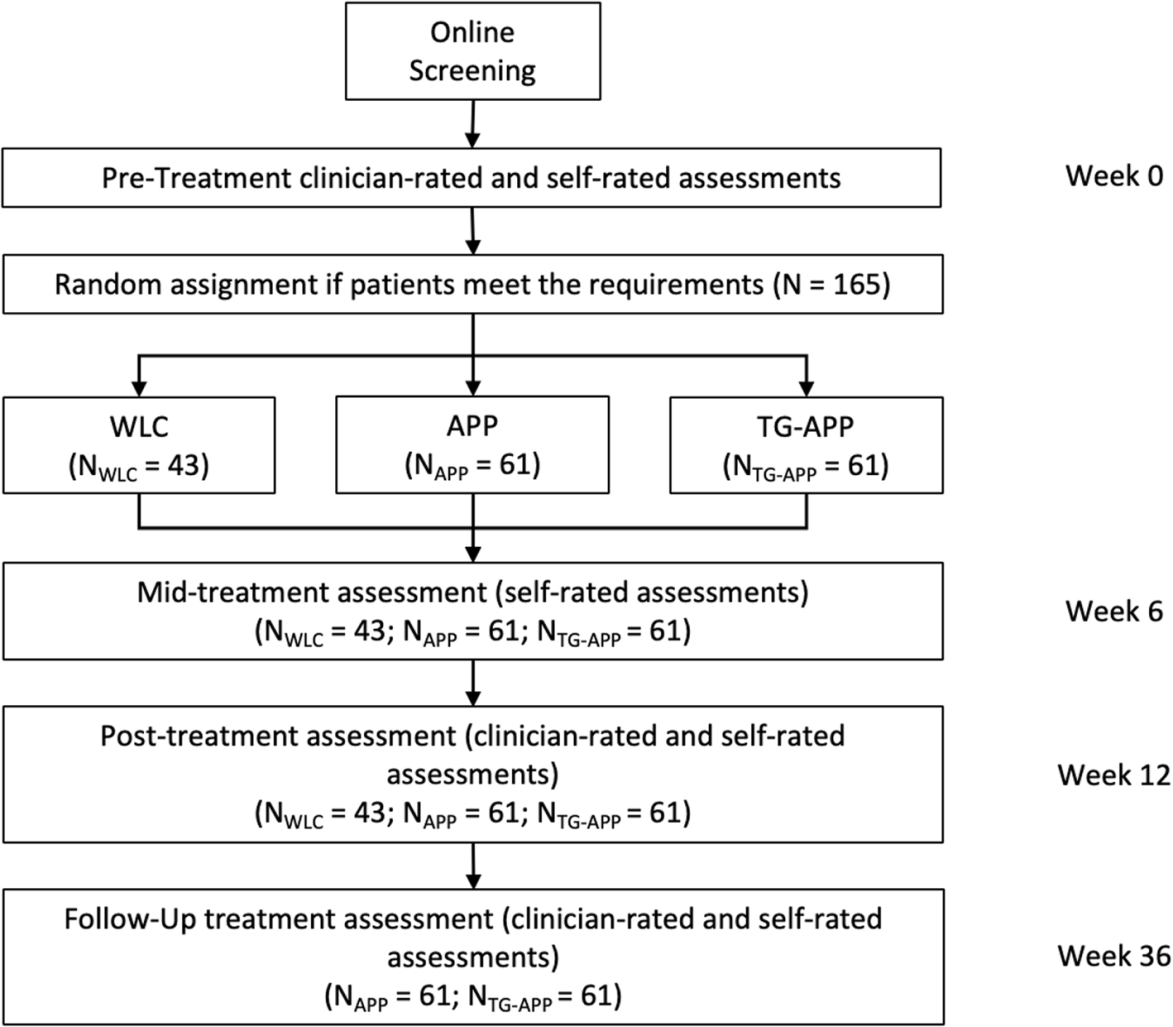
Study flowchart

We based our power analysis on an iterative simulation of increasing sample sizes with starting values of *n* = 30 for the control condition and *n* = 50 for each of the treatment conditions, increasing stepwise by one participant per condition. The variance of the random intercepts and residual variance was set to 0.5 respectively [24]. Because missing values due to the dropout of participants were planned to be imputed by the jump to reference method (J2R) [25], the reference being the control condition, we reduced our expected effected sizes by multiplying them with the term (1—dropout-rate). Given a dropout rate of 26% at post-treatment evaluation in the study by Stolz et al. [16] and a pooled post-treatment dropout rate of 26.2% in a meta-analysis of studies using smartphone apps by Torous et al. [26], we chose a conservative estimate of 30% post-treatment dropout for the APP condition and multiplied the expected effect size of 0.5 by 0.7, equaling to *d* = 0.35. For the TG-APP condition, we expected a lower post-treatment dropout rate of 20% due to the additional guidance by a therapist and multiplied the expected effect size of 0.8 by 0.8, equaling to *d* = 0.64. Based on 500 simulations of the linear mixed model fitted with the expected effect sizes, we obtained the power of finding a statistically significant interaction of group × time based on a likelihood ratio test, compared to finding only main effects, and the 95% confidence interval for this estimate, for increasing sample sizes. We located the minimum sample size in our results after which the lower bound of the 95% confidence interval did not fall below 80% power, given *α* = 0.05. This condition was met at *n* = 41 for the control group and *n* = 61 for both treatment conditions (power estimate = 84%, 95% confidence interval = 80.0, 87.0). In addition to our study purposes, the app provider intends to use the results of the pilot study and the main study for the licensing process of the app as a digital health product. For this licensing process, a separate statistical analysis will be conducted comparing only the APP condition with the WLC condition, using a *t*-test for statistical analysis. A corresponding additional sample size calculation revealed the need to recruit 43 subjects in the wait-list control group (WLC). To establish conformity across the planned analyses we adjusted the number of participants for WLC upward to *n* = 43, resulting in a total sample size of *N* =165 (see also Fig. 1).

As a robustness check, we additionally conducted a power analysis based on the simulation of a 3 × 3 interaction (group × time) that included the estimated effect sizes at *t*_3_ (follow-up assessment), for which we obtained a highly similar sample size.

### Procedure

The study will be conducted in Germany at the outpatient psychotherapy unit of the Clinical Psychology department of the Goethe University of Frankfurt as well as the outpatient psychotherapy unit of the Clinical Psychology department of the Technische Universitaet Dresden and two further CBT training centers in Dresden. For reporting purposes, we followed the SPIRIT reporting guidelines [27].

In the first step, all persons that register for participating in the study will be screened using an online self-report assessment. If participants exceed the cut-off of 25-points or find themselves within the criteria for social anxiety disorder, they are referred to another online self-assessment and then invited for an interview with a clinical rater. Beforehand, they will be informed about the study and provided with written informed consent (either personally or via email if necessary). Interviews to determine the patient's formal diagnosis are conducted by blinded and independent psychologists (with at least a master’s degree) who have been trained in the appropriate interviews. Eligible patients are then randomly assigned to one of three groups (WLC, APP, TG-APP). Participation in each of the groups lasts 3 months. Participants in the WLC group then have the option of using the app if they wish. The outcome measures will be assessed by blinded and independent raters at baseline, post-treatment (3 months after start), as well as 6 months after the post-treatment interview (follow-up). Furthermore, participants will complete self-report questionnaires online at baseline, at mid-treatment (at 6 weeks), post-treatment (at 12 weeks), and follow-up (at 36 weeks; see Fig. 1 for the study flowchart). Interviewers will remind patients to fill out the self-report questionnaires and if necessary, patients will be contacted by the study coordinator to complete the missing questionnaires.

Prior to the start of the study, we will conduct a pilot study to improve usability and evaluate the mobile application and the applicability of therapist-guided video therapy and the videoconferencing tool.

### Ethical issues

The study was approved by the Ethics Committee of the Department of Psychology at Goethe University Frankfurt. If post-trial care is required (e.g., in case of serious adverse effects), this will be provided by the outpatient clinics in Frankfurt and Dresden, and participants will be referred to a hospital or psychiatric clinic if necessary.

### Recruitment

Advertisements for participation include print media (e.g., brochures or text in print media), posts on social media, and outreach to mental health professionals, inpatient and outpatient facilities, and general practitioners to identify appropriate participants.

### Eligibility criteria

Inclusion criteria:

- Current diagnosis of Social Anxiety Disorder
- Written informed consent before the start of the study
- Age: 18 to 65 years
- Possession of smartphone
- Familiarity with using apps

Exclusion criteria:

- Acute suicidality
- Active substance abuse or dependence
- Severe medical conditions (e.g., chronic cardiovascular disease)
- Severe depression
- Psychotic disorder
- Bipolar disorder
- Borderline personality disorder
- Current psychotherapeutic treatment
- Current psychopharmacological treatment
- No proficient skills in the German language

Furthermore, patients may be withdrawn from the study for the following reasons:

- Request of patient
- Post-hoc observation of criteria for exclusion
- Threat to patient’s mental or physical health due to participating in the study

### Allocation and randomization

Randomization is performed using a randomization list in which, based on 165 randomly generated numbers sorted by size, each of the available options is assigned according to the planned sample size (WLC = 43, APP = 61, TG-APP = 61). The list is then sorted by sequential number. A study coordinator who is not involved in the diagnosis or treatment process will enter the subjects into the randomization list according to the order of inclusion. The result is communicated to the patients via email and remains hidden from the clinical evaluators. The email to the participant contains the result and, if the person belongs to one of the two intervention groups, all the necessary information to use the app.

### Treatment

The app offers an adaptation of scientifically proven techniques in the treatment of SAD based on Clark and Wells [4] and is intended for use as a mobile-based intervention. This intervention is designed to reduce the wait time for treatment and help therapists reduce the number of sessions while maintaining or increasing the effectiveness of treatment. The general idea is that using the app will facilitate the treatment process for both the patient and the therapist, as patients can practice the protocolized treatment steps in their environment and therapists can then incorporate these experiences into therapist-led change processes. The entire treatment program will last three months.

Clark and Wells’ model [4] aims to change the factors that maintain social anxiety. Treatment includes derivation of a model and behavioral experiments to test the effect of safety behaviors and self-focused attention, attention training, video feedback, further behavioral experiments, and cognitive restructuring. Based on this model and its German adaptations by Stangier et al. [28] and Hoyer and Härtling [29], the treatment includes the following modules:

- Module 1—Learning and Understanding: The goal of this module of the app is to provide the user with key information about SAD and to create an individualized model based on Clark and Wells’ approach. The information used to derive the model is based on patients’ self-reports of anxiety-provoking situations. The model is developed in a stepwise manner, prompted by feedback from the app.
- Module 2—Attention Training: The goal of the second module is to change self-focused attention. The training uses recorded audio exercises that train the ability to focus attention on external stimuli.
- Module 3—Behavioral experiments: This key module of the app consists of four phases. Each phase includes specific types of behavioral experiments, as well as various forms and questions for planning the experiments and recording the results and key findings.
  The first phase provides an experiment on how increasing or decreasing safety behaviors and self-focused attention affects anxiety. The experiment is supplemented by video feedback, in which patients are asked to record themselves in a simulated social situation (e.g., a presentation) and subsequently correct dysfunctional beliefs about their appearance or behavior.
  The second phase involves testing the dysfunctional beliefs that emerge in real social situations while simultaneously reducing safety behaviors and directing their attention outward.
  The third phase focuses on testing beliefs about the assumed negative impact of supposedly embarrassing behaviors on how they are evaluated by others (e.g., “If I pause during a presentation, I will appear awkward, and others will think I am an idiot”). Patients are instructed to explicitly demonstrate certain critical behaviors (e.g., pausing during a presentation) and to question the validity of their (possibly erroneous) beliefs.
  The fourth phase involves integrating behavioral experiments into daily life by continually testing SAD-related beliefs that interfere with personal goals.

In the therapist-guided app condition (TG-APP), use of the app is accompanied by eight therapist-guided video sessions. Based on the manual by Stangier et al. [28], the therapist assists patients in conducting behavioral experiments and provides a better understanding of the underlying processes. The goals of the sessions are as follows:

- First session: establishing a therapeutic alliance, exploring the symptoms and giving an introduction to the app
- Second session: answering questions based on the individualized model of the app, deriving a second cognitive model by exploring safety behaviors and establishing motivation for change
- Third session: performing a video recorded behavioral experiment to modify safety behaviors and self-focused attention
- Fourth session: analyzing the recorded video to test dysfunctional self-evaluation (video feedback)
- Fifth session: conducting a therapist-guided behavioral experiment testing a dysfunctional belief
- Sixth session: conducting a therapist-guided behavioral experiment deliberately showing critical behavior and testing the evaluation by others
- Seventh session: motivating the patient to implement behavioral experiments into the everyday life and debriefing of previous behavioral experiments
- Eighth session: relapse prevention (summary of helpful techniques, pursuing personal goals, cognitive interventions for strengthening the self-esteem)

### Therapists

Therapists will be recruited mainly from the outpatient units of the Universities of Dresden and Frankfurt. All therapists must be either licensed psychotherapists or in advanced post-graduate CBT training (at least 1.5 years of training). Study-specific training will focus on how to use the app, how to conduct and analyze behavioral experiments, and how to use techniques of cognitive restructuring. Therapists are required to apply the TG-APP procedures with a pilot patient to gain experience and expertise in this particular form of blended therapy. All treatments will be supervised by psychotherapists with at least five years of clinical experience under their state licensure to ensure adherence to intervention protocols.

### Outcomes

#### Primary outcome measure

We defined the clinician-rated Liebowitz Social Anxiety Scale (LSAS) [21, 22], as the primary outcome measure, a measure widely considered the gold standard in controlled studies on SAD. The LSAS is designed as an interview to assess fear and avoidance in 24 different social situations (rated on a 4-point scale from “not at all” or “never” to “severe” or “most of the time”). The LSAS demonstrated good internal consistency (Cronbach’s *α* = 0.96), validity and treatment sensitivity [30].

#### Self-rating scales

As the secondary outcome measure for SAD, we use the Social Phobia Inventory (SPIN) [31]. The SPIN is a self-report questionnaire designed to assess the amount of discomfort in different situations during the last week. The total score can range between 0 and 68. Its psychometric properties are satisfactory [32, 33]. The Mini-SPIN [34], a short version with 3 items, is used as a screening instrument.

We also use the German version of the Social Cognitions Questionnaire (SCQ, German version: SPK) [35, 36], which lists 22 beliefs typically related to social anxiety (e.g., “People will see I’m nervous”) that are rated on two scales: the mean thought frequency, ranging from 1 (“thought never occurs”) to 5 (“thought always occurs”) and a mean belief rating, ranging from 0 (“I do not believe this thought”) to 100 (“I am completely convinced this thought is true”).

Symptoms of depression and general psychological distress will be assessed using the Beck Depression Inventory Fast Screen (BDI-FS) [37] and the 18-item version of the Brief Symptom Scale (BSI-18) [38]. The BDI-FS is the abbreviated version of the widely used Beck’s Depression Inventory (BDI-II) [39], which consists of 7 items, including for example “Sadness,” rated on a 4-point-scale. The questionnaire shows good reliability and validity ratings [40]. The BSI-18 is the short version of the original BSI [41], a questionnaire measuring general psychological distress rating items like “feelings of loneliness” on a scale from 0 (“not at all”) to 4 (“a lot”). The German version of the BSI-18 shows satisfactory internal consistency and validity [38]. Sensitivity to rejection in social situations are assessed with the Social Pain Questionnaire (SPQ-5) [42]. The questionnaire consists of 5 items (e.g., “If I have the feeling that a colleague pulls back from me, I feel rejected”) rating from 0 (“describes me perfectly”) to 4 (“not at all”). Initial psychometric properties show good reliability and validity [42].

To assess the quality of life, we will use specific domains of the WHO-QOL-BREF [43] that include the overall quality of life, as well as psychological quality, and quality of social relationships. The WHO-QOL-BREF asks for ratings on varying 5-point scales from 1 (e.g., “not at all” or “very dissatisfied”) to 5 (e.g., “completely” or “very satisfied”) for items like “How satisfied are you with your sleep?”. We excluded the domains of physical health and environment because they are not relevant to our study. The questionnaire shows good to excellent reliability and good validity [44].

To measure the amount of distress caused by the mental illness, we used the Pain and Disability Index (PDI) [45]. It consists of 7 different categories of life (like “Social Activity”), each of which is rated on a scale from 0 (“no disability”) to 10 (“total disability”). Reliability and validity seem adequate [46]. Additionally, the Anticipatory and Consummatory Interpersonal Pleasure Scale (ACIPS) [47] will be used to measure the pleasure patients experience in interpersonal situations with 17 items. It demonstrated good reliability and validity [47].

To assess if individual learning styles influence treatment effects, we included Kolb’s Learning Style Inventory (LSI) [48], where participants have to match different sentences (e.g., “when I learn…”) with four proposed items (e.g., “… I like to be active”) that are arranged on a scale from 1 (“describes me the worst”) to 4 (“describes me best”), so that each item for each sentence has a unique number from 1 to 4. It demonstrates satisfactory internal reliability and evidence for the suggested internal structure [49].

Treatment satisfaction will be measured by using the Client Satisfaction Questionnaire (CSQ) [50], which we adapted slightly to match the use of the app. This 8-item questionnaire uses questions like “How satisfied are you with the amount of help you received?“. It showed satisfying internal consistency and concurrent validity [51, 52]. Social anxiety and social approach behavior will be measured using a modified version of the Social Phobia Weekly Summary Scale (SPWSS) [53], which will be assessed by participants weekly (for the APP and the TG-APP condition) and at pre, mid, post and follow-up assessments (for all conditions). The SPWSS covers ratings of anxiety, avoidance, self-focused attention, anticipatory worry, post-event rumination, and social approach behavior. We added questions asking for feelings of pleasure and pursuit of personal goals and changed the scale to a 0–100 scale. The SPWSS is sensitive to treatment effects and has good internal consistency [53].

Finally, we will also assess negative side effects which are specific to patients with SAD. Drawing on the results of Boettcher et al. [19], we developed a brief (7-item) scale with items covering the negative side effects that were considered most relevant by a group of experts (see Additional file 1). The items are rated on a scale from 0 (“do not agree at all”) to 5 (“fully agree”). Please note that the descriptive list of possible side effects is preliminary and may be slightly revised after the pilot study. As the list contains an open item, responses to this item will be evaluated after the pilot study and inclusion of further side effects in the list is possible should they be mentioned frequently.

#### Clinician-rated measures

The interview will include the Structured Clinical Interview for DSM 5 (SCID-5) [54] conducted by clinician independent raters. Psychometric properties showed good reliability and specificity [55].

To screen for borderline personality disorder (BPD), we use a set of 5 questions suggested by Wongpakaran et al. [56] that screen for symptoms of BPD based on the DSM-V and a Rasch analysis. It showed good quality for assessing BPD [56]. If screening indicates that BPD may be present, we use the appropriate category of the SCID-5 to check for the other criteria of BPD.

We further use the Clinical Global Impression (CGI) [57] modified for patients with SAD. The CGI-Severity scale provides information about the current severity of social phobic symptoms which are rated on a 7-step scale from “normal” or “not ill at all” to “among the most severely ill patients” by a clinician. The CGI-Improvement scale is a 7-point scale rating the change in symptom severity from ‘improved by a lot’ to “a lot worse.” The use of the CGI, as a measure for symptom-specific improvement for patients with SAD, is supported by adequate psychometric properties [58] and its practicality.

**Fig. 2:**
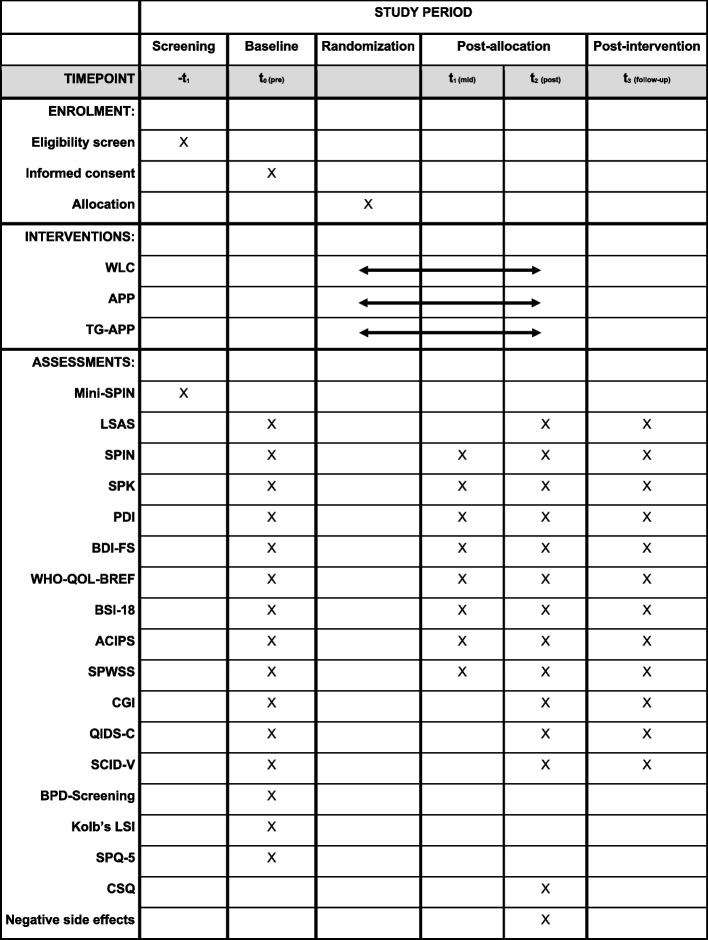
Schedule of enrolment, allocation, interventions, and assessments

Finally, the Quick Inventory of Depressive Symptomatology (QIDS-C) [59] will be applied, a 16-item rating instrument for the assessment of depressive symptoms by an independent interviewer, which showed acceptable internal consistency and treatment sensitivity [60]. Figure 2 gives an overview of the measures and assessments used in this study.

### Statistical methods

#### Sample

All analyses will be conducted in the intent-to-treat (ITT) sample, including all participants that were randomized into one of the three groups.

#### Primary analysis

For our primary analysis, we plan to fit a linear mixed model with random intercepts for participants in the R package lme4 [61]. We take the *z*-standardized values of the Liebowitz Social Anxiety Scale (LSAS) as the outcome variable, and time and treatment condition as categorical predictors, for which we specify an interaction term. The estimated effects of the predictor variables indicate the change in LSAS from baseline at week 0, which we choose as the reference category, to post-treatment.

#### Secondary analyses

As secondary analyses, we will evaluate treatment effects (from baseline to post-treatment, as described in hypothesis 2a and from post-treatment to follow-up, as described in hypothesis 2c) on quality of life, as measured by the WHO-QOL-BREF, interpersonal pleasure, as measured by the ACIPS, further SAD-related symptoms, as measured by the SPIN and the CGI, social phobic cognitions, as measured by the SPK, symptoms of depression, as measured by the BDI-FS and the QIDS-C, general psychological distress, as measured by the BSI, and limitations in everyday life, as measured by the PDI. We will also assess adverse effects measured by the self-developed adverse effect scale. Moreover, we will compare posttreatment LSAS scores with scores at follow-up, as stated in hypothesis 1c, using the linear mixed model described earlier.

To control for moderating effects on decreases in social anxiety symptoms, as indicated in hypothesis 3, we include the scores of the individual learning style, as measured by Kolb’s LSI and experienced pain in social situations, as measured by the SPQ-5, as predictors in the model and specify three-way interactions with treatment conditions and time for all these factors. We will conduct a similar analysis to test whether social approach behavior measured by the SPWSS has a mediating effect on social anxiety symptoms, as we hypothesized in hypothesis 4. We will test all interactions by comparing the full to a reduced model without the respective interaction term via likelihood ratio tests (LRTs) [62] and remove all non-significant interactions from the final model.

To evaluate effects between two conditions at a given time, e.g., between the APP and TG-APP conditions at post-treatment, as indicated in hypothesis 1b and 2b, we will calculate the statistical significance of pairwise differences in LSAS based on the linear mixed model using the R package “emmeans” [63].

We will fit all measures as outcome variables in linear mixed models including the same predictors as in the primary analysis and add the average weekly time participants spent using the app and the number of behavioral experiments conducted as additional explanatory predictors.

Besides that, we will analyze and compare responder rates between the different treatment conditions at post-treatment using a chi-squared test. Response to treatment will be defined as a 29% score decrease of the clinician-rated LSAS from baseline to post-treatment according to Glischinski et al. [64].

#### Drop-out analyses

We define as “drop-out” any participant who did not complete the post-treatment measurement. Systematic differences in dropout rates between the three groups will be analyzed by a Fisher exact test. In addition, differences between participants and drop-outs in terms of sociodemographic and clinical variables will be considered.

#### Interim analysis

In case of a rejection of the licensing process by the inspecting federal institute for drugs and medical devices, an interim analysis of the treatment effect of the APP condition on outcomes of the main study (LSAS, WHO-QOL-BREF, PDI) could turn out to be necessary. Such interim analysis will be blinded, that is, performed by different researchers than the respective research teams of the study and after reaching a sample size of *n* = 30 for the APP and for the WLC group at post-treatment (based on a one-tailed power analysis simulation in R for a mean difference between two independent groups with unequal variance and effect size of *d* = 0.35, a standard deviation of outcome variables of 0.7 for both groups and 80% power). To account for multiple hypothesis testing, the alpha error level will be adjusted according to Pocock [65]. The interim analysis will not affect the continuation of the study.

## Discussion

Based on a recent meta-analysis, there is evidence that internet- or app-based interventions have moderate effect sizes in the treatment of SAD [12]. There is also preliminary evidence from previous studies that internet-based cognitive behavioral therapies (ICBTs), based on the model of Clark and Wells [4], may achieve large effect sizes [15, 16, 66]. These findings are consistent with results demonstrating the superiority of CBT based on Clark and Wells’ model in regular face-to-face therapy [5]. In our study, we plan to extend these findings by conducting an RCT comparing the efficacy of a mobile app based on Clark and Wells’ model with the efficacy of the app plus additional therapist-guided sessions delivered via video and a wait-list control group.

We hypothesize that the addition of therapist-guided sessions will result in larger effect sizes than using the app alone. In agreement with Stott et al. [15], we assume that contact with a therapist has a critical impact on engagement in specific CT interventions, particularly behavioral experiments. Similar to exposure-based treatments, the presence of a therapist assists the patient in overcoming avoidance, shifting attention outward, and identifying and discontinuing safety behaviors and ensures that feared negative evaluation is challenged. Therefore, therapist-guided sessions can prevent the underuse of behavioral experimentation, an important effective component of CT according to Clark and Wells’ model.

To summarize, our results will demonstrate (a) the efficacy of a new app-based intervention using the Clark and Wells approach in another well-designed and preregistered independent trial and (b) whether additional face-to-face therapy is beneficial to patients in terms of increased symptom reduction of SAD compared to app use alone. The results will be critical for service delivery and treatment planning for social anxiety disorder.

### Organizational structure

#### Coordinating center and trial steering committee

The present study is a bicentric study which is designed and coordinated in a cooperation between the Goethe University Frankfurt and the Technische Universitaet Dresden. The study team has weekly meetings. There is no additional steering committee.

Support for the trial is provided by:

- Principal investigator: provides supervision for the treatment, has medical responsibility of the patients, designs and plans the study
- Study coordinator: coordinates study procedures, responsible for administrative tasks, reviews study progress
- Study therapists: responsible for the implementation of the therapies
- Clinical raters: responsible for conducting independent clinical evaluations with respect to outcome criteria and inclusion and exclusion criteria

#### Data monitoring committee

There is no data monitoring committee and no independent auditing process. The quality of the data is assured by the blinded clinical raters and there are no known serious adverse effects. If serious adverse effects occur, they will be reported to the principal investigator, who will then interview the patient in question and refer them to a psychiatry if needed. Adherence to therapeutic measures is ensured by regular supervision by the principal investigators. Completeness of data will be regularly verified by the study coordinators.

#### Data management and confidentiality

Questionnaires will be answered online. The screening is the only time participants enter personal contact information. In the subsequent assessments and questionnaires, participants are identified by a random computer-generated code. Other than the study coordinators, only therapists and clinical raters know the participant’s name. The anonymized data is stored in an encrypted folder on a protected research server at Goethe University Frankfurt. There is also a separate encrypted list that contains only contact information and the code so that participants can be contacted if needed. This list is deleted after the study, so that only the codes with the assigned data remain. The program R is used to analyze the data. The results of the study will be published only in peer-reviewed journals. The informed consent form will be kept in a locker at the outpatient clinics of the universities of Frankfurt and Dresden.

#### Relevant concomitant care and interventions that are permitted or prohibited during the trial

There is no concomitant care additional to the intervention groups that we already described. If there are medical conditions of the patients, that need to be treated right away, patients will be send to a physician. If patients start a different psychotherapy while being part of the study, they will drop out.

## Trial status

Recruitment began in July 2022. The current protocol is version 2 of 16 December 2022. There have been no patients enrolled into the trial so far (16 September 2022). Approximate date of completed recruitment: 31 December 2023.

## Supplementary Information

**Additional file 1.** Items for the negative side effects questionnaire.

## Data Availability

The data used in this study, including for example, the R code for randomization, are available from the corresponding authors upon reasonable request.

## Abbreviations

LSAS: Liebowitz Social Anxiety Scale
SPIN: Social Phobia Inventory
SPK: Fragebogen zu sozialphobischen Kognitionen
PDI: Pain and Disability Index
BDI-FS: Beck Depression Inventory - Fast Screen
WHO-QOL-BREF: World Health Organization Quality of Life
BSI-18: Brief Symptom Inventory - 18-item version
ACIPS: Anticipatory and Consummatory Interpersonal Pleasure Scale
SPWSS: Social Phobia Weekly Summary Scale
CGI: Clinical Global Impression
QIDS-C: Quick Inventory of Depressive Symptoms
SCID-V: Structured Clinical Interview for DSM-5
Kolb’s LSI: Kolb’s Learning Style Inventory
SPQ-5: Social Pain Questionnaire
CSQ: Client Satisfaction Questionnaire
