# Supplementary material for: Evaluation of a smartphone application for self-help for patients with social anxiety disorder: a randomized controlled study—SMASH
Source: Trials. 2023 Mar 1;24:154. doi: 10.1186/s13063-023-07168-5 (PMC9974392; doi:10.1186/s13063-023-07168-5)
Supplement: Supplementary file 1 — Additional file 1. Items for the negative side effects questionnaire. [file 13063_2023_7168_MOESM1_ESM.pdf]

Items for the negative side effects questionnaire

1.	I feel that my symptoms have worsened as a result of using the app.
2.	New symptoms have arisen while using the app.
3.	I have had more strained relationships with significant others (e.g., family, partnership, friends, colleagues) since using the app.
4.	I felt worse because I felt like I wasn't making progress with the app.
5.	I felt worse because I felt that the app made the full extent of my problems clearer.
6.	I felt like the procedure was useless because I would only get anywhere with a therapist.
7.	I felt that doing exercises was being watched or seen through by others and felt stigmatized (labeled as a patient) as a result.
	Are there any other negative effects you experienced? ______ ______ ______
